# Cross‐Sectional Analysis of Research and Non‐Research Payments From the Medical Device Industry to Healthcare Professionals and Organisations in Japan in 2022

**DOI:** 10.1111/jep.70066

**Published:** 2025-03-26

**Authors:** Anju Murayama

**Affiliations:** ^1^ School of Medicine Tohoku University Sendai Japan

**Keywords:** conflicts of interest, ethics, health policy, Japan, medical device industry payment

## Abstract

**Rationale:**

Financial relationships between healthcare professionals (HCPs), healthcare organisations (HCOs) and the medical device industry in Japan may lead to conflicts of interest. Limited transparency in these relationships has raised concerns regarding their potential influence on clinical decision‐making and patient care.

**Aims and Objectives:**

This study aimed to assess the scope and magnitude of financial payments from medical device companies to HCPs and HCOs in Japan, focusing on publicly disclosed data from the 2022 fiscal year.

**Methods:**

A cross‐sectional analysis of publicly available payment disclosures from 169 medical device companies, including all members of the Japan Federation of Medical Devices Associations (JFMDA) and several major non‐JFMDA affiliated companies, was conducted. Descriptive statistics were calculated to summarise payment data.

**Results:**

Of the 169 medical device companies, 32.5% (55 companies) did not disclose payment information and 6 companies disclosed payment records in aggregated amounts with their group companies, leaving 108 companies for analysis. In total, $245.2 million in payments were made in 2022, with only 23.9% ($58.7 million) to research and development. Of the total, 34.6% ($84.9 million) was allocated to non‐research payments to HCOs for sponsoring HCOs’ activities, and 19.6% ($49.0 million) were paid for lecturing and consulting fees, typically to individual HCPs. More than 80% of companies allocated a greater share of payments to non‐research activities than to research.

**Conclusion:**

This study reveals substantial financial relationships between medical device companies and HCPs/HCOs in Japan, predominantly for non‐research purposes. Improved transparency regulations, including a uniform, government‐run database, are necessary to better oversee these financial interactions with medical device industry in Japan.

## 1 Introduction

Financial relationships between healthcare professionals (HCPs), healthcare organisations (HCOs) and industry are pervasive, extending beyond research and development to various aspects of clinical care, including industry‐sponsored medical education, product marketing and the provision of free samples. However, these relationships introduce conflicts of interest (COIs) for HCPs and HCOs, which may compromise their professional judgement and ultimately jeopardise patient care [1, 2]. A substantial body of evidence, particularly from high‐income countries such as the United States, several European nations and Japan, underscores the potential undue influence of financial relationships between the pharmaceutical industry and HCPs and HCOs on the quality of care and professional decision‐making [3, 4, 5].

Since 2013, the Japan Pharmaceutical Manufacturers Association (JPMA), the largest trade association in Japan's pharmaceutical industry, has voluntarily encouraged its member companies to disclose financial contributions to HCPs and HCOs [3]. Previous studies utilising this payment data have revealed widespread and substantial financial relationships between HCPs, HCOs and the pharmaceutical industry in Japan [3, 6, 7, 8]. For instance, from 2017 to 2021, the pharmaceutical industry provided a total of $86.2 million to the 15 most influential internal medicine societies in Japan [3]. Moreover, over 90% of the physicians involved in developing clinical guidelines received payments from pharmaceutical companies [7, 8, 9].

Similarly, the Japan Federation of Medical Devices Associations (JFMDA), the largest trade association for major medical device companies in Japan, introduced transparency guidelines in 2012, requiring its members to voluntarily disclose payments made to HCPs and HCOs [10]. While some illegal financial relationships between HCPs and medical device companies have been investigated in recent years, little is known about the overall extent of payment disclosures by medical device companies or the magnitude of financial relationships between HCPs, HCOs and the medical device industry in Japan. This study aimed to examine the scope and magnitude of disclosed payments from all medical device companies to HCPs and HCOs in Japan.

## 2 Methods

This cross‐sectional study analysed publicly available payment information disclosed by medical device companies as of August 2024. The latest data available pertained to payments made to HCPs and HCOs during the 2022 fiscal year. Given that the study involved an analysis of nonhuman participants using publicly available data, it was exempt from institutional review board oversight in accordance with the Ethical Guidelines for Medical Research Involving Human Participants at Tohoku University.

The JFMDA transparency guidelines require all member companies to disclose payment information on their respective websites. Payment records are classified into five major categories: (A) research and development expenses, (B) academic research grants, (C) manuscript writing fees, (D) information provision‐related expenses and (E) other expenses. Each of these categories contains further subcategories, with categories B–E categorised as non‐research‐related payments. Detailed definitions and examples of each category are provided in Supporting Information S1.

To identify payment disclosures, I first compiled a list of all JFMDA member companies from the association's website and created an Excel file. Non‐profit organisations were excluded from the study, as were pharmaceutical companies affiliated with both the JFMDA and the JPMA, which disclosed payments in accordance with JPMA transparency guidelines. Additionally, given that some major medical device companies (e.g. Smith & Nephew, Zimmer Biomet, Carl Zeiss, Kyocera and Intuitive Surgical) are not JFMDA members, I specifically searched the websites of these companies for disclosure information. Non‐JFMDA companies that were highly likely to make large payments to HCOs and HCPs in Japan were identified through my literature review of previous studies reporting payments to HCOs and HCPs in European countries and the United States [11, 12, 13, 14], as well as my research experience and interest in the Japanese context. For each company, I conducted a Google search using the keywords ‘company name’ + ‘transparency guidelines (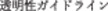 in Japanese)’ to determine whether payment information was disclosed. If no relevant results were found, I manually searched the company's website using relevant terms such as ‘transparency’, ‘disclosure’, or ‘conflict of interest’. If payment disclosure information could not be identified within 10 min, the company was classified as non‐disclosing. For companies that disclosed payment information, I extracted the total amounts by category, the disclosure period and the unit of payment amounts for the 2022 fiscal year.

The data were analysed using descriptive statistics. For companies that disclosed monetary values, the total amount and per‐company payments were calculated for each payment category. All the payment values were converted into US dollars using the 2022 average monthly exchange rate of 131.4981 Japanese yen per $1 USD, as reported by the International Financial Statistics of the International Monetary Fund [15].

## 3 Results

As of August 2024, I assessed the payment disclosure status of 169 medical device companies (Supporting Information S2). Of these, 55 companies (32.5%), all affiliated with the JFMDA, did not disclose payment information. As six subsidiaries' payment records were consolidated into their respective parent companies' totals, these six companies were excluded, leaving 108 companies for analysis.

Of the 108 companies, two explicitly stated that no payments were made to HCPs and HCOs in 2022. There was considerable variation in the reporting periods (Supporting Information S3). The total payments made by the remaining 106 companies in 2022 amounted to $245,234,446 (Table 1). Of this total, 34.6% was allocated to academic research grants [B], typically provided to HCOs for supporting professional activities such as conferences, meetings and research grants. Manuscript writing fees [C] accounted for 19.6% ($49.0 million) of the total, typically paid to individual HCPs. Only 23.9% ($58.7 million) of the payments were allocated to research and development expenses [A]. The median total payment (interquartile range) per company was $741,833 ($165,494–$2,460,772), with $299,668 ($91,940–$1,116,126) for academic research grants [B] and $112,263 ($32,010–$399,650) for manuscript writing fees [C].

**Table 1:** Payments from medical device companies to healthcare professionals and organisations in 2022.

Payment category	Total payment amounts, $	Number of companies making payments (*n* = 108), n	Payment amounts per company, $			
			Median payments (interquartile range)	Mean payments (standard deviation)	Minimum payments	Maximum payments
Research and development expenses (category A)	58,659,819 (23.9%)	83 (76.9%)	194,414 (48,391–806,278)	706,745 (1,343,638)	11	9,411,307
Academic research grants (category B)	84,907,806 (34.6%)	95 (88.0)	299,668 (91,940–1,116,126)	893,766 (1,478,051)	760	9,258,653
Manuscript writing fees (category C)	48,039,975 (19.6%)	101 (93.5)	112,263 (32,010–399,650)	475,643 (1,021,886)	84	6,476,477
Information provision‐related expenses (category D)	44,935,767 (18.3%)	89 (82.4)	87,165 (20,951–475,770)	504,896 (1,071,917)	373	6,293,710
Other expenses (category E)	8,691,082 (3.5%)	96 (88.9)	28,249 (4,068–113,027)	90,532 (154,868)	46	953,560
Overall	245,234,446 (100%)	106 (98.2%)	741,833 (165,494–2,460,772)	2,313,533 (4,164,266)	404	26,452,878

The largest subcategory of payments was cosponsorship expenses for academic conferences [B4], accounting for 15.5% ($38.0 million) of the total, followed by scholarship donations [B1] (14.2%, $34.9 million), lecture and other meeting expenses [D1] (13.5%, $33.1 million) and lecturer fees [C1] (12.5%, $30.6 million) (Supporting Information S4).

Medtronic made the largest payments in 2022 ($26.5 million), followed by Nipro ($17.5 million), Terumo ($15.8 million), Johnson & Johnson ($15.6 million) and Abbott Medical ($15.3 million) (Table 2). Medtronic also had the highest total for non‐research payments [categories B–E] at $23.0 million. Of the 108 companies, 87 (80.5%) made more payments to HCPs and HCOs for non‐research purposes than for research and development [category A].

**Table 2:** Top 10 medical device companies making the largest total payments in 2022.

Company name	Total aggregate amounts, $	Total amounts of non‐research payments [categories B–E], $	Total amounts of research payments [category A], $
Medtronic Japan	26,452,877	22,982,399	3,470,478
Nipro	17,519,769	8,108,462	9,411,307
Terumo	15,819,854	12,614,592	3,205,262
Johnson & Johnson	15,577,984	15,033,077	544,907
Abbott Medical Japan	15,259,953	12,477,843	2,782,111
Boston Scientific Japan	11,085,759	8,698,469	2,387,291
Stryker Japan	7,728,641	7,497,643	230,998
Canon Medical Systems	7,072,142	2,688,180	4,383,962
Edwards Lifesciences	7,057,432	5,982,946	1,074,486
Olympus	6,428,813	4,987,495	1,441,318

## 4 Discussion

This is the first comprehensive analysis of payments made to HCPs and HCOs by more than 100 major medical device companies in Japan for 2022. The findings revealed that medical device companies paid more than $245.2 million to HCPs and HCOs in a single year, with the majority allocated to non‐research activities such as sponsorships, product promotion at medical societies and consulting fees for individual HCPs. Notably, most companies allocated more payments to non‐research activities than to research and development. More than one‐third of medical device companies did not disclose their payments to HCPs and HCOs, even though they affiliated with JFMDA. These findings provide critical insights into the financial relationships between HCPs and HCOs and the medical device industry in Japan.

The substantial non‐research payments to HCPs and HCOs in Japan are consistent with previous research [11, 16, 17]. The total non‐research payments made by medical device companies in Japan were higher than those reported in other high‐income countries. For example, while the medical device market in Japan ($28.97 billion) is only 1.6 times larger than that of the United Kingdom ($18.56 billion) [18], Japanese medical device companies reported $84.9 million in academic research grants (category B) in 2022, which is approximately 7.2 times higher than the average annual non‐research payments to HCOs reported in the United Kingdom ($11.8 million per year during 2017–2019) [11, 19]. These findings suggest potential differences between the two countries in the regulatory framework for industry payments, the strategic objectives of companies, the role of financial relationships in health systems and the influence of HCOs in the supply and use of medical devices. Further research is warranted to explore these country‐level differences and their implications for healthcare policy and practice.

Moreover, this analysis revealed that significant payments were made for lecturing and consulting services, typically to individual HCPs. Previous research reported that medical device companies in the United States made a total of $281.3 million ($70.3 million per year) to individual physicians for lecturing and consulting services from 2014 to 2017, with most payments going to specialists in neurosurgery, orthopedic surgery, urology, cardiology and general surgery [16]. In Japan, medical device companies paid a total of $48.0 million for manuscript writing fees, which include lecturing, manuscript writing and consulting fees [category C] in 2022, primarily to individual HCPs. Given the difference in the number of active physicians between the United States (about 949,600) and Japan (about 323,700), the population‐adjusted amount of non‐research payments for lecturing and consulting may be higher in Japan than in the United States.

Despite the substantial size of non‐research payments to HCPs and HCOs in Japan compared to those in other high‐income countries, a more comprehensive and detailed examination of the underlying causes and factors behind these payments in Japan was constrained by limitations in the regulatory framework and the reporting requirements under the JFMDA disclosure guidelines. First, payment records are disclosed by individual medical device companies on their respective websites, and a uniform, comprehensive payment database for medical device companies in Japan does not currently exist. In contrast, governments and trade organisations in other high‐income countries, including the United States and several European nations, have developed such databases, making payments from medical device companies to HCPs and HCOs publicly available [11, 16]. Therefore, in Japan, payment records are not categorised by individual HCPs and HCOs, limiting the ability to analyse payments at the recipient level. Second, the payment records lack detailed information regarding the context and recipients of the payments, such as the associated product names, the disease areas linked to the payments, and the specialties of the recipient HCPs—details that are available in other countries, such as the United States [20, 21, 22]. Third, the accuracy of payment records disclosed by medical device companies is not verified by third parties, which raises the possibility of underestimating the financial relationships between HCPs, HCOs and medical device companies in Japan. For instance, in a recent bribery case involving a medical device company, Nihon Kohden failed to accurately disclose payments to anesthesiologists on its website, as the payments were made through a third party [23]. As a result, the overall size of payments from medical device companies to HCPs and HCOs may be underreported. Fourth, the JFMDA transparency guidelines do not cover certain types of payments to HCPs and HCOs, including royalties and licensing fees, while these payments are covered in other countries, such as the United States [16, 22, 24]. Finally, as this study only considered all JFMDA members and several non‐JFMDA member companies, there could be financial relationships between HCPs and HCOs and medical device companies that were not covered in this study.

In conclusion, there are substantial financial relationships between HCPs and HCOs and medical device industry mainly for non‐research purposes in Japan. To improve transparency in financial relationships between HCPs and HCOs and medical device industry, more rigorous and transparent regulations including introduction of government‐run uniform database are necessary in Japan.

## Author Contributions

Anju Murayama contributed to study design, data collection, resource, software, formal analysis, visualisation and study administration. Anju Murayama is the guarantor of this study, accepts full responsibility for the work and/or the conduct of the study, had access to the data, and controlled the decision to publish.

## Ethics Statement

As this study was a retrospective analysis of publicly available data and met the definition of nonhuman subjects research, no institutional board review and approval were required. This study followed the Strengthening the Reporting of Observational Studies in Epidemiology (STROBE) guideline.

## Consent

The author has nothing to report.

## Conflicts of Interest

The author received grant from Tohoku University School of Medicine outside of this study.

## Supporting information

Supporting information.

## Data Availability

All data used in this study is publicly available from each company affiliated with the Japan Federation of Medical Devices Associations (JFMDA). The datasets generated during and/or analysed during the current study are available from the corresponding author on reasonable request.
