# Supplementary material for: Cross‐Sectional Analysis of Research and Non‐Research Payments From the Medical Device Industry to Healthcare Professionals and Organisations in Japan in 2022
Source: J Eval Clin Pract. 2025 Mar 26;31(2):e70066. doi: 10.1111/jep.70066 (PMC11938807; doi:10.1111/jep.70066)
Supplement: Supplementary file 1 — Supporting information. [file JEP-31-0-s001.docx]

Supplemental Material 1. Definitions and examples of payment categories

| Payment category | Definitions and examples |
| --- | --- |
| Category A. Research and development expenses, etc. | Research funds incurred for research and investigations conducted in accordance with public regulations, including the Clinical Research Act and the GCP/GVP/GPSP Ministerial Ordinance based on the Pharmaceuticals and Medical Devices Act, as well as the various pertinent guidelines. |
| A1. Specific clinical research expenses | Research costs incurred under the terms of the contract for the purpose of conducting clinical research as defined in the Clinical Research Act in Japan. |
| A2. Research expenses based on ethical guidelines | Research funds provided to medical institutions, etc., for research conducted under the “Ethical Guidelines for Medical and Biological Research Involving Human Subjects” in Japan. |
| A3. Non-clinical research expenses | Research funds provided to medical institutions, etc., for research other than specific clinical research, research based on ethical guidelines, clinical trials (clinical studies), and post-marketing surveillance, etc., such as so-called “basic research.” |
| A4. Expenses of clinical trial for marketing authorization | Research funds for clinical trials, post-marketing clinical studies, adverse event/infection case reports, and post-marketing surveillance conducted under pharmaceutical regulations such as GCP/GVP/GPSP. This category also includes research funds provided for investigator-initiated clinical trials. |
| A5. Post-marketing clinical trial expenses | Research funds for post-marketing clinical trials. |
| A6. Failure/infectious case reporting fee | Research funds associated with adverse event or infection case reports. |
| A7. Post-marketing surveillance expenses | Research funds for post-marketing surveillance studies. |
| A8. Other R&D related expenses | Research funds that do not fall into the seven categories mentioned above are classified into this category. This category includes expenses related to meetings held for research and development. |
| Category B. Academic research grants | This category includes scholarship donations, general donations, and contributions to support academic conferences and similar events for the purpose of promoting academic activities and research. In general, these funds include non-research payments to healthcare organizations such as academic medical societies, universities, academic centers, research institutions, hospitals, clinics, specific medical departments and laboratories.  This funds may also include academic societies and other organizations to support the holding of academic conferences, co-sponsorship fees, and similar activities. |
| B1. Scholarship donations | Research grants or endowed chairs provided to institutions conducting research (universities, research institutions, etc.)  Endowed chairs will be disclosed under the category "Scholarship Donations," including the name of the endowed chair and the total number of donations and amounts provided for the fiscal year. It is not necessary to explicitly indicate that the funds were for an endowed chair.  The usages of donations are not pre-specified by the companies in a majority of the scholarship donations. |
| B2. General donations | Donations provided to support the overall activities of organizations or institutions.  Donations to research institutions, foundations, etc., will be disclosed individually under "General Donations." If medical institutions receive donations through foundations, their names will be disclosed if identifiable, otherwise, the foundation's name will be disclosed. |
| B3. Academic society contributions | Donations provided to support the holding of academic conferences and other academic meetings.  Contributions to academic societies made via foundations will be disclosed by listing the academic society’s name and the amount paid to the foundation. The foundation’s name does not need to be disclosed. |
| B4. Co-sponsorship expenses for academic conferences, etc. | Expenses paid to co-sponsor seminars and other events in collaboration with academic societies, including costs paid to academic societies for holding academic conferences.  Co-sponsorship includes fees for seminars, advertising on academic society websites and abstracts, exhibition booth fees, sponsorship fees, and related expenses.  All funds for jointly sponsored meetings with medical organizations will be disclosed here. For jointly sponsored meetings with medical institutions, expenses will be disclosed under “D. Information provision-related expenses.” |
| B5. Other payments | This includes financial contributions, such as one-time donations or support for events or activities not classified under academic conferences or research-related activities (e.g. sponsorship of academic awards and research grants organized by professional medical societies). |
| Category C. Manuscript writing fees, etc. | This category includes fees paid as compensation for outsourced services such as lectures, manuscript writing, supervision, and other consulting work related to providing information on the proper use of the company’s medical devices. The companies must disclose the detailed names of payment recipient for this category.  The two-tier disclosure system, where detailed information is only provided upon request, is not permitted for disclosing “C. Manuscript writing fees, etc.” All information must be fully disclosed without limitations. |
| C1. Lecturer fees | Payments made to healthcare professionals for honoraria paid to speakers, moderators, or panelists at lectures, research meetings, etc.  “C. Manuscript writing fees, etc.” payments will, in principle, be made to the individual contractor, and the name of the institution, department, position, and individual will be disclosed. If payment is made through the individual's affiliated medical institution, the same details (institution, department, position, individual name) will still be disclosed. |
| C2. Manuscript writing and supervision fees | Fees for manuscript writing and supervision for publications related to medical or biomedical engineering topics.  If payments under “C. Manuscript writing fees, etc.” are made directly to the contractor’s affiliated medical institution, the institution's name, number of payments, and total amount will be disclosed, but the individual contractor's name will not be disclosed. |
| C3. Consulting and other outsourcing fees | Fees for consulting services related to product development, business strategies, or guidance on the proper use of medical devices.  If payments are made to an entity other than the contractor’s affiliated medical institution, the name of the entity, the name of the contractor, and the name of the affiliated institution will be disclosed along with the number of payments and the total amount. |
| Category D. Information provision-related expenses | This category includes expenses necessary for providing proper and safe usage information of the company’s medical devices to healthcare professionals, such as lecture fees, mock practical training sessions, and briefing session costs.  Companies will disclose the aggregate total amounts of payments for this category on an annual basis. |
| D1. Lecture and other meeting expenses | Costs related to organizing lectures and research meetings focused on the company’s medical devices or relevant topics in medicine and biomedical engineering, aimed at healthcare professionals. |
| D2. Briefing fee | Costs related to organizing briefings (such as department meetings) and mock practical training sessions aimed at healthcare professionals to explain the proper use of the company’s medical devices. |
| D3. Medical and medical engineering literature provision expenses | Costs related to providing literature, educational materials, and patient education materials related to the company’s medical devices. |
| Category E. Other expenses | This category includes hospitality expenses and other costs incurred as part of customary social practices. Companies will disclose the aggregate total amounts of payments for this category on an annual basis. |

Legend: This table summarizes the disclosure requirements endorsed by the Japan Federation of Medical Devices Associations (<https://www.jfmda.gr.jp/wp/wp-content/uploads/2022/11/%E5%8C%BB%E6%A9%9F%E9%80%A3%E3%83%BB%E9%80%8F%E6%98%8E%E6%80%A7%EF%BC%A7%EF%BC%AC%E3%80%90%E8%A7%A3%E8%AA%AC%E3%80%912022.10%E6%94%B9%E5%AE%9A%E5%B7%AE%E6%9B%BF%E7%89%88.pdf>).

Supplemental Material 2. Flowchart of selection of eligible medical device companies

Supplemental Material 3. Disclosure characteristics

| Variables | Number of company |
| --- | --- |
| Total number of companies included in the payment analysis | 108 |
| JFMDA membership |  |
| JFMDA affiliated companies | 93 (86.1%) |
| Non-JFMDA member companies | 15 (13.9%) |
| Time period covered by the 2022 disclosure record |  |
| From April 2022 to March 2023 | 38 (35.2%) |
| From January 2022 to December 2022 | 32 (29.6%) |
| Unspecified in the company’s disclosure system | 20 (18.5%) |
| From October 2021 to September 2022 | 9 (8.3%) |
| September 2021 to August 2022 | 2 (1.9%) |
| From July 21, 2022 to July 20, 2023 | 2 (1.9%) |
| From July 2022 to June 2023 | 2 (1.9%) |
| Other time period | 3 (2.8%) |
| Monetary units of disclosed payment amounts |  |
| Per 1 Japanese yen (equal to about $0.008) | 101 (93.5%) |
| Per 1,000 Japanese yen (equal to about $7.6) | 7 (6.5%) |
| Disclosure of payment records at the individual recipient level for categories A, B, and C |  |
| Disclosed payment records at the individual recipient level, in accordance with the JFMDA guidelines | 104 (96.3%) |
| Disclosed only aggregate total amounts for each major category | 2 (1.9%) |
| No payments were made to HCPs and HCOs in 2022 | 2 (1.9%) |

Payment value was converted into US dollar value, using the average monthly exchange rate of 131.4981 Japanese yen per $1.

Supplemental Material 4. Categories of Payments from Medical Device Companies in Japan, 2022

| Payments by category | Payment amounts (%), $ |
| --- | --- |
| Research and development (A) | 58,697,842 (23.9) |
| Specific clinical research expenses (A1) | 5,895,487 (2.4) |
| Research expenses based on ethical guidelines (A2) | 18,990,953 (7.7) |
| Non-clinical research expenses (A3) | 13,387,851 (5.5) |
| Expenses of clinical trial for marketing authorization (A4) | 12,195,506 (5.0) |
| Post-marketing clinical trial expenses (A5) | 1,022,929 (0.4) |
| Failure/infectious case reporting fee (A6) | 355,108 (0.1) |
| Post-marketing surveillance expenses (A7) | 2,369,073 (1.0) |
| Other R&D related expenses (A8) | 4,120,450 (1.7) |
| Subcategory unspecified by companies | 360,485 (0.1) |
| Academic research grants (B) | 84,907,805 (34.6) |
| Scholarship donations (B1) | 34,935,139 (14.2) |
| General donations (B2) | 7,337,011 (3.0) |
| Academic society contributions (B3) | 3,849,480 (1.6) |
| Co-sponsorship expenses for academic conferences, etc. (B4) | 38,048,949 (15.5) |
| Other expenses (B5) | 343,123 (0.1) |
| Subcategory unspecified by companies | 394,103 (0.2) |
| Manuscript writing fees, etc. (C) | 48,039,975 (19.6) |
| Lecturer fees (C1) | 30,605,034 (12.5) |
| Manuscript writing and supervision fees (C2) | 1,770,619 (0.7) |
| Consulting and other outsourcing fees (C3) | 13,955,122 (5.7) |
| Other expenses (C4) | 112,263 (< 0.1) |
| Subcategory unspecified by companies | 1,596,937 (0.7) |
| Information provision-related expenses (D) | 44,935,766 (18.3) |
| Lecture and other meeting expenses (D1) | 33,053,328 (13.5) |
| Briefing fee (D2) | 9,399,268 (3.8) |
| Medical and medical engineering literature provision expenses (D3) | 1,787,119 (0.7) |
| Other expenses (D4) | 241 (< 0.1) |
| Subcategory unspecified by companies | 695,810 (0.3) |
| Other expenses (E) | 8,691,082 (3.5) |

Legend: Values represent financial allocations across different categories of healthcare industry expenditure. All monetary values are in US dollars. Percentages are rounded to one decimal place. Values less than 0.1% are denoted as "< 0.1". "Subcategory unspecified by companies" rows indicate amounts where detailed categorization was not provided by companies.
